# Adsorptive removal of Rhodamine B dye from aqueous solution by using graphene–based nickel nanocomposite

**DOI:** 10.1016/j.heliyon.2021.e06851

**Published:** 2021-04-22

**Authors:** Usha Jinendra, Dinesh Bilehal, B.M. Nagabhushana, Avvaru Praveen Kumar

**Affiliations:** a Department of Chemistry, Karnatak University, Dharwad 560008, Karnataka, India; b Department of Chemistry, MSRIT, Bengaluru 560054, Karnataka, India; c Department of Applied Chemistry, School of Applied Natural Science, Adama Science and Technology University, P.O. Box: 1888, Adama, Ethiopia

**Keywords:** Reduced graphene-nickel nanocomposite (RGO–Ni), Rhodamine B dye, SEM, XRD, Adsorption kinetics

## Abstract

In this work, reduced graphene oxide-nickel (RGO–Ni) nanocomposite is synthesized. X-ray diffraction (XRD), scanning electron microscopy (SEM) and SEM–EDS (Energy Dispersive X-Ray Spectroscopy) are used to study the crystalline nature, morphology and elemental composition of the RGO–Ni nanocomposite, respectively. As synthesized RGO–Ni nanocomposite is used to develop selective adsorptive removal of Rhodamine B (RhB) dye from the aqueous solution. The experiments have been performed to investigate RhB uptake via RGO–Ni nanocomposites which include, contact time (60 min), initial dye concentration (50 mg/100 ml), adsorbent dosage (0.5 mg) and pH 8 of dye solution. The equilibrium concentration is determined by using different models namely, Freundlich, Langmuir and Tempkin. Langmuir isotherm has been fitted well. Langmuir and Tempkin equations are determined to have good agreement with the correlation coefficient data. The kinetic study concluded that RhB dye adsorption follows with the pseudo-second-order kinetic model. Further, adsorption mechanism of RGO–Ni is proposed which involves three steps. The synthesized adsorbent is compared with the other adsorbents in the literature and indicates that RGO–Ni nanocomposite used in this study shown better results for a particular adsorption capacity than polymeric, natural and synthetic bioadsorbents. The regeneration and reusability experiments suggest RGO–Ni nanocomposite can be used for many numbers of times for purification/adsorption.

## 1 Introduction

Textile industries generate the most liquid effluent due to the vast amount of water involved in the dying process [1]. Synthetic dyes are used in a wide variety of items like clothing, food, and leather accessories. Between 10 to 15% of the unused dyes are coming out as effluents [2]. Apart from un-pleasant aspects of wastewater that contains dye, it's presence in naturally available water bodies including, rivers and streams may cause major damage to aquatic life due to its toxic nature. And some dyes which are reported as carcinogenic and also non-biodegradable [3].

**Figure 1:**
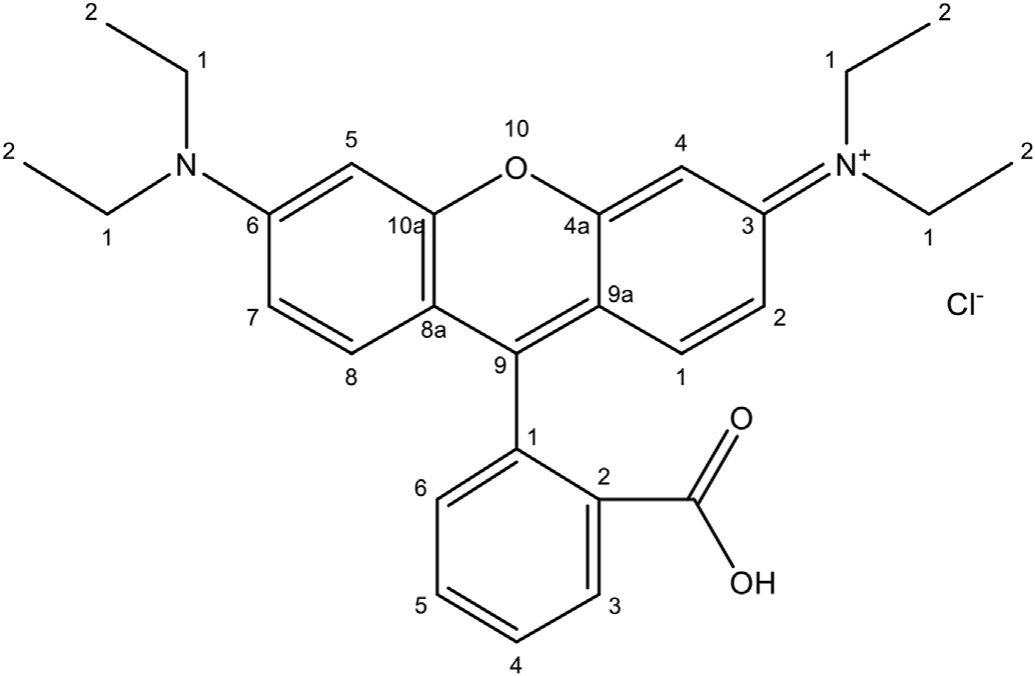
Structure of RhB dye.

At the moment, over 9000 distinct dyes are used, each one referring to a distinct chemical application class. A number of the chemical and physical treatments, like, precipitation, adsorption, etc. are mostly used to get free from these harmful pollutants from water. Many of the developed methods have some drawbacks [4]. The removal of dyes in a productive way is the most important work for environmental scientist. In photochemical degradation of dye, which is based on electron-hole pairs that are obtained, react to water as well as oxygen molecules to generate hydroxide radicals, superoxide anions which increase oxidizing power and thereby to be employed in degradation of numerous industrial dye compounds [5]. One among is Rhodamine B (RhB) dye (Figure 1) which has two molecular forms of zwitter ionic and cationic.

Cationic dyes are treated as toxic colorants which may induce health effects such as cancer, skin irritation, allergic dermatitis and mutations. The use of activated charcoal adsorption is better suited for the treatment of wastewater containing dye. Focusing on the productive way by evaluating the capacity to adsorb heavy metals and dye pollutants present in the wastewater [6, 7]. Adsorption plays a crucial role in the removal of dyes. Adsorption kinetics and isotherm in a series of batch modes give the useful information for the calculation of effectiveness of adsorbent-adsorbate system [8, 9].

Taking these factors into account, there is a need to develop a new and modified technique for the treatment of waste water containing dyes. A wide range of graphene-based nanocomposites used in water purification in many cases as well as maximum removal of toxic waste without any waste disposal problem has been reported [10]. Literature survey reveals that Suryakanti Debata et al. have done the synthesis of graphene based nickel nanocomposite with RhB dye removal where they focused on synthesis, characterization and studied the preliminary dye removal degradation but not studied in detail [11, 12]. However, present work describes detailed study about batch studies, adsorption isotherms, kinetics, mechanism, comparison with other adsorbents using RhB dye and also regeneration of the adsorbent. In another case, along with synthesis and characterization and preliminary degradation of RhB dye removal, they have verified the usage of composite for electrode material as super capacitors [13]. The graphene based nanocomposites are also used in devices like dye sensitized solar cell [14], enhanced X-ray photon response [15], enhanced performance of light-controlled conductive switching [16], and photocatalytic activity in semiconductor [17].

In order to improve the adsorption and dye degradation efficiency by using graphene-based nanomaterials, along with co-catalysts, noble metal particles, metallic, non-metallic doping and with hydrogels have been used [18]. Even though many researchers reported on the dye degradation of RhB, our work using graphene based nickel nanocomposite (RGO–Ni) showed better adsorption efficiency than adsorption for removing RhB dye compared to that of other adsorbents like TiO_2_ and graphene TiO_2_ [19], CuS/PVA nanocomposite [20], ZnO–MnO_2_ [21], and other self-assembled GO nanostructures from various styles [22, 23, 24, 25].

Graphene (G) is tightly packed into a two-dimensional (2D) "honeycomb lattice". Graphene is used in an excessive application such as power storage, adsorption and dye degradation properties [26, 27, 28]. In this quick evaluation, the latest studies advance in graphene-nickel nitrate prepared with sonication, are provided with the purpose of extending the adsorption of RhB dye in detail. The RhB dye (Figure 1) was chosen as a representative organic toxic waste in order to assess the adsorption kinetics of the pollutant onto a graphene-based nickel nitrate nanocomposite under various experimental conditions. The kinetic data were analyzed and various models were used to match the experimental data. This may be useful in large scale study and practical applications in the dyeing wastewater treatment [29].

## 2 Experimental

### 2.1 Materials

In the laboratory, graphene and reduced graphene oxide (RGO) were synthesized using an improved Hummer's process. Rhodamine B dye (C_28_H_31_ClN_2_O_3_) (AR Grade, 99%), hydrogen peroxide (H_2_O_2_) (98%), potassium permanganate (KMnO_4_) (AR Grade, 99%), sulfuric acid (H_2_SO_4_) (98%), Ortho phosphoric acid (o-H_3_PO_4_) (98%), methanol (98%) and hydrazine hydrate (H_6_N_2_O) (98%) were purchased from Sigma-Aldrich. The double distilled water was used for this entire study.

### 2.2 Graphene oxide preparation

**Figure 2:**
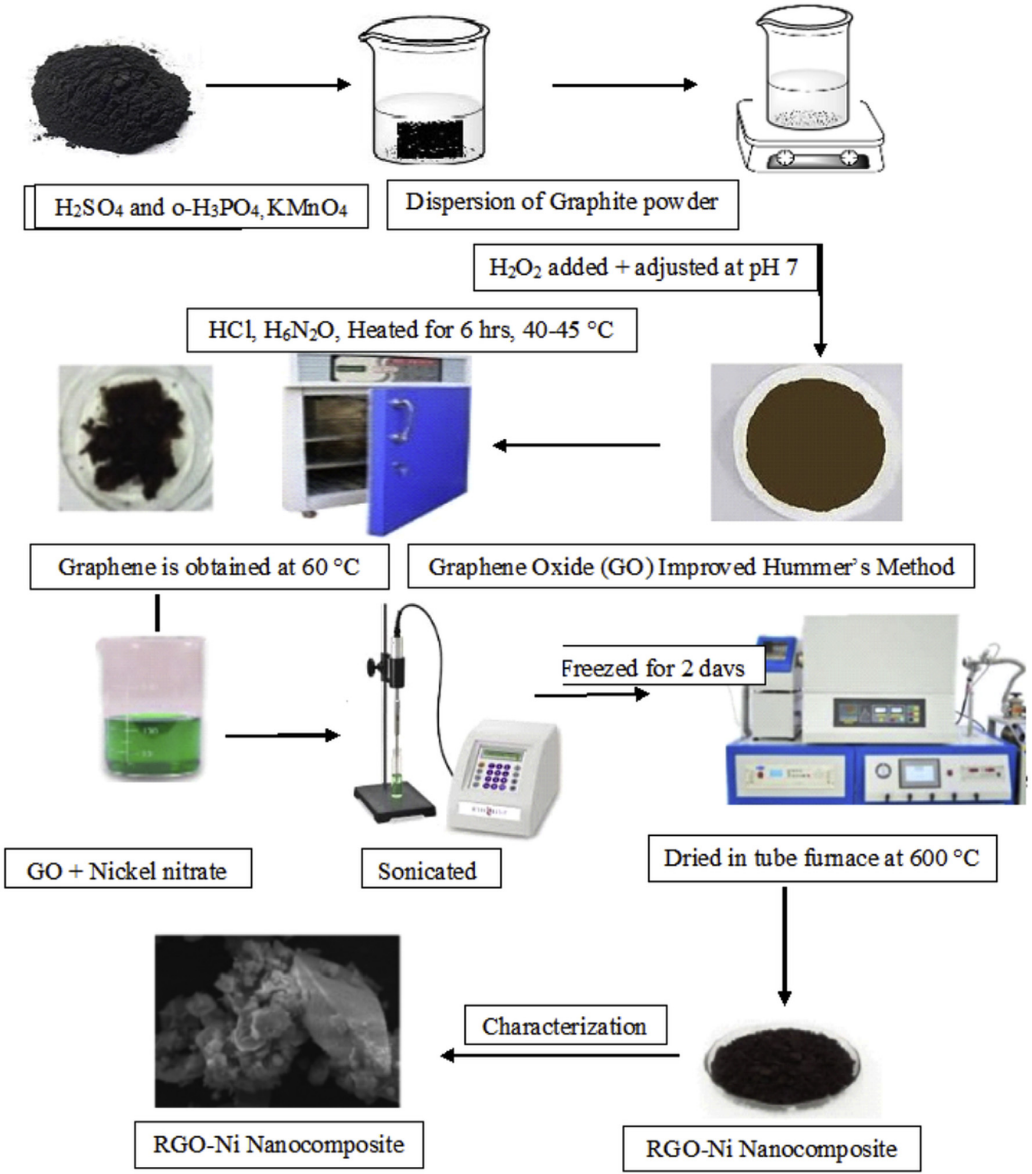
Flow chart of the preparation of RGO-Ni nanocomposite by Hummer's method.

Much interest is to obtain graphene and graphene oxide preparation by improved Hummers' method. The first step involves oxidation. In this oxidation step, the natural graphite was dissolved in H_2_SO_4_ and o-H_3_PO_4_ acidic mixture under high speed stirring in an ice bath. The temperature was then steadily raised to 30–50 °C by adding KMnO_4_. And the temperature was kept at 50 °C for approximately 20 h with constant stirring. After 20 h, the reaction mixture was mixed with ice-cold deionized water and then mixing with H_2_O_2_ at room temperature for around 3 h. The solution was adjusted to pH 7 using distilled water. HCl was used for the washing process to move out the metal ions. The obtained RGO was dried at 60 °C. The second step involves reduction. In this step, required amount of graphene oxide (GO) was dissolved in methanol. To this 10 ml of hydrazine hydrate (H_6_N_2_O) was added with continuous stirring. The solution was heated up to 40–45 °C under constant mixing for 6 h. This product obtained was filtered, washed, and then dried at 60 °C to get graphene (Figure 2).

### 2.3 Preparation of reduced graphene oxide-nickel (RGO-Ni) nanocomposite

Figure 2 shows the flow chart for the preparation of RGO–Ni nanocomposite. By dissolving the nickel nitrate in 20 ml of deionized water and adding 20 ml of RGO solution (4 mg/ml) vigorously stirring, the necessary ratio of RGO to nickel nitrate was obtained. A 10/1 mass ratio of RGO/Ni was maintained. The solution was sonicated, and the obtained solution was kept in a freezer (~18 °C), and kept in vacuum at the temperature below 0 °C for 2 days (Figure 2). The obtained product was heated from the room temperature (RT) to 600 °C at the rate 10 °C/min in a tube furnace and held at 600 °C temperature for 3 h.

### 2.4 Preparation of dye solution for batch study

Every day, stock solutions of RhB dye (300 mg/L) were prepared. Further, working standard solutions were prepared daily from the stock solutions through sufficient dilution with deionized water. Adsorption studies were performed in a series of batch mode. Adsorbent (weighed) and 50 mL of dye solution were taken in a 100 ml beaker and then mixed on a magnetic stirrer. The stirring speed was kept at 250 rpm and the temperature was set to 25 °C (room temperature). The pH of the solution was changed by adding HCl or alkali solution and then measured with a pH meter. By using centrifuge and filtration, the adsorbent particles were removed from the solution. Change in the absorbance (Eq. (2)) of the test sample was studied in different time intervals. At the conclusion of this study, the concentration of the dye was measured using the Elico SL 159 ultraviolet–visible (UV–Vis) spectrophotometer. The adsorption capacity q_e_ can be calculated using Eq. (1):

(1) q_e_ = [(C_0_–Ce)/V] × M

(2) % Adsorption = (C_i_–C_e_)/C_i_

where: q_e_ amount of dye adsorbed in mg per gram of adsorbent.

C_0_ and Ce (mg/L) are initial and equilibrium dye concentrations in the liquid phase, respectively.
C_i_ and C_e_ are initial and equilibrium concentration of RhB (mg/L), respectively.
V is the volume of mass of adsorbent.
M is the mass of adsorbent.

### 2.5 Characterization of RGO–Ni nanocomposite

**Figure 3:**
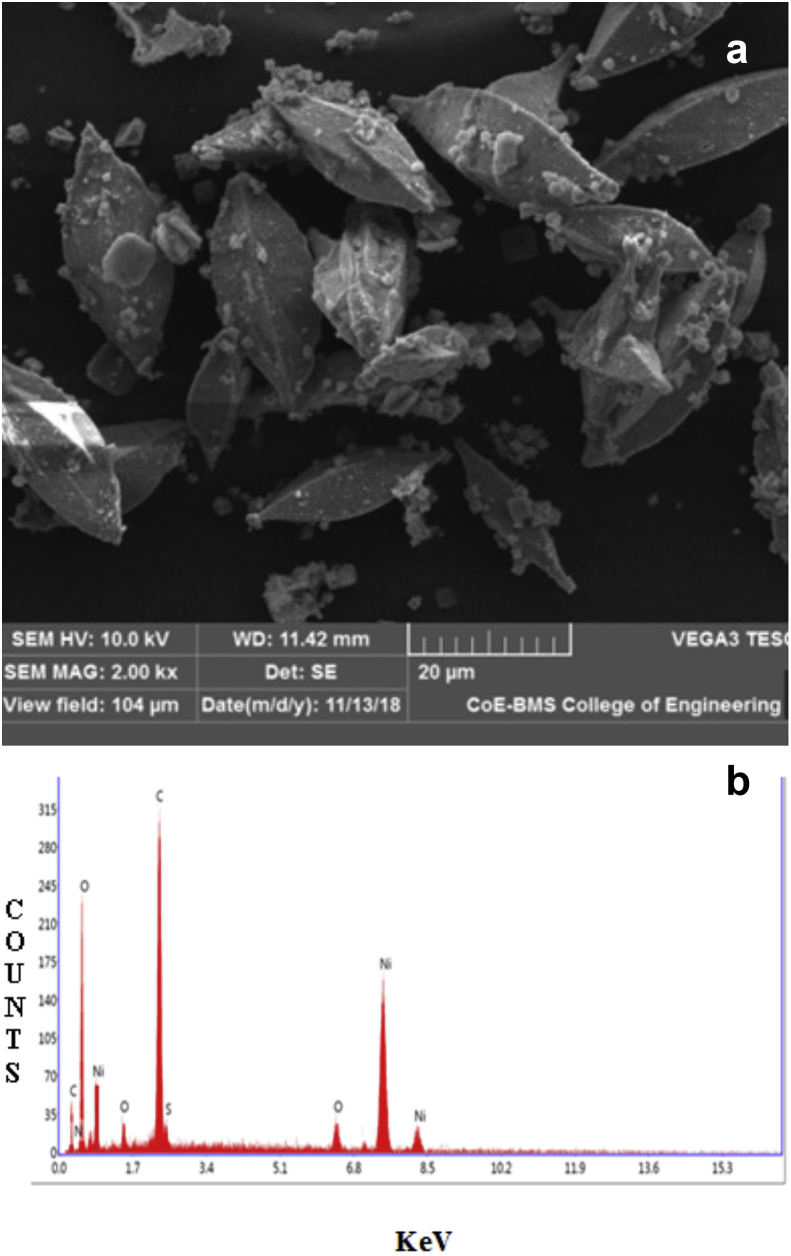
(a) SEM micrograph and (b) EDS spectrum of RGO-Ni nanocomposite

**Figure 4:**
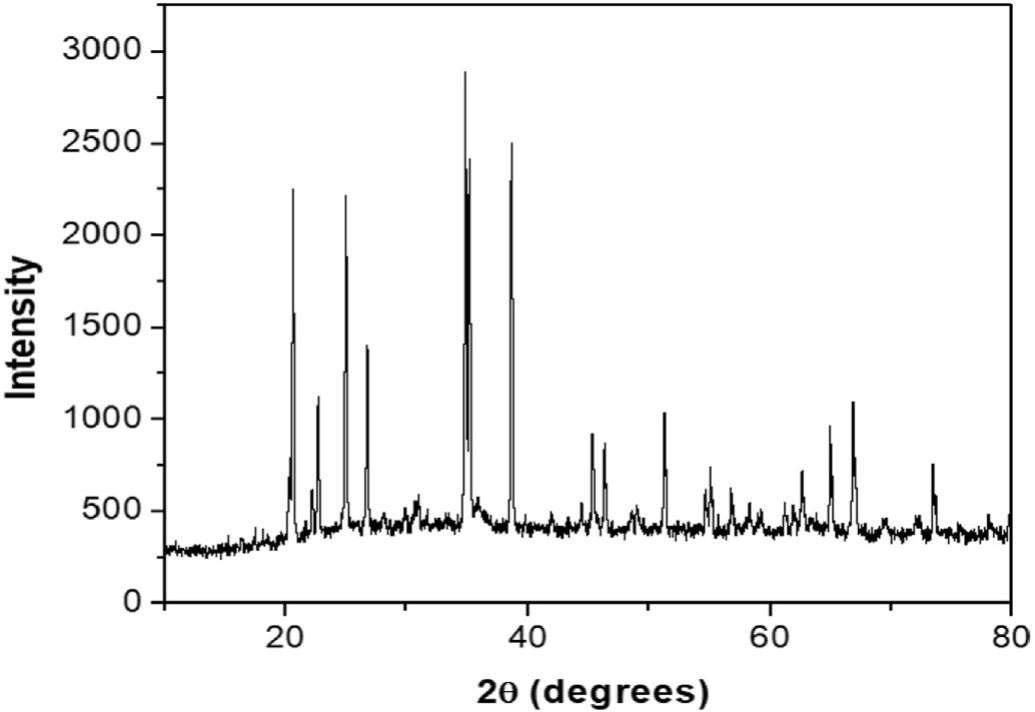
XRD pattern of RGO-Ni nanocomposite

The scanning electron microscopy (SEM) picture of RGO–Ni nanocomposite was shown in Figure 3a, which displays diamond shape material having porous surface. By this Figure, it is concluded that RGO–Ni nanocomposite have suitable morphology for RhB adsorption. Energy Dispersive X-Ray Spectroscopy (EDS) results reveal that elements such as oxygen (38.51%), carbon (34.39%), sulphur (1.49%), aluminum (1.01%), nitrogen (1.84%), iron (0.67%) and Nickel (13.49%) are contained in RGO–Ni nanocomposite sample (Figure 3b). Further, the nanocomposite composed of aluminum and iron impurities which confirm that they are obtained from heating of tube furnace to 600 °C. X-ray diffraction (XRD) data of RGO–Ni nanocomposite was collected between scattering angles (2θ) of 10–80° at a scanning rate of 2° min^−1^ from the Siemens D-5000 diffractometer operating with Cu-Kα radiation (λ = 1.54056 Å). The XRD pattern was shown in Figure 4 which indicates that the RGO–Ni nanocompositeis crystalline in nature with clear characteristic peaks and matched well as JCPDS file 98-001-6741. The XRD pattern of RGO–Ni nanocomposite could be concluded that the original graphite powder was almost completely oxidized. However, the oxygen-incorporated functional groups of graphene oxide couldn't be completely got cleared off through Hummers reduction. A small diffraction peak is persisted in the XRD pattern of RGO–Ni nanocomposite which is characteristic of graphene oxide at 35^o^ approximately, as well a broad peak of the graphite at 34^o^. The characteristic diffraction peaks of graphite from 49^o^ to 54^o^ were due to the short-range order of graphene.

### 2.6 BET surface area measurement

At a bath temperature of -196.3 °C, the total surface area of the synthesized graphene–based nickel nanocomposite was calculated using nitrogen adsorption/desorption isotherms on a Brunauer–Emmett–Teller (BET) surface area analyzer (ASAP 2020). Using the N_2_ adsorption/desorption isotherm results, the total surface areas of the given samples were calculated using the BET multi-point and single-point methods. Prior to the analysis, the graphene–based nickel nanocomposite was evacuated under vacuum for an entire night at 150 degrees Celsius to clean the pores. The BJH method, a technique for calculating pore size distribution using the Kelvin equation and DH method, used to calculate the pore volume data. The Dubinin–Radushkevich (DR) method was used to determine the micropore volumes of the samples. The BJH model was used to evaluate the pore size distribution. Surface area (m^2^/g):18.0, pore volume (cm^3^/g): 0.029, pore size (nm): 6.3 and micropores area (m^2^/g): 10.57 summarize all BET surface area measurement parameters.

## 3 Results and discussion

### 3.1 Effect of contact time

**Figure 5:**
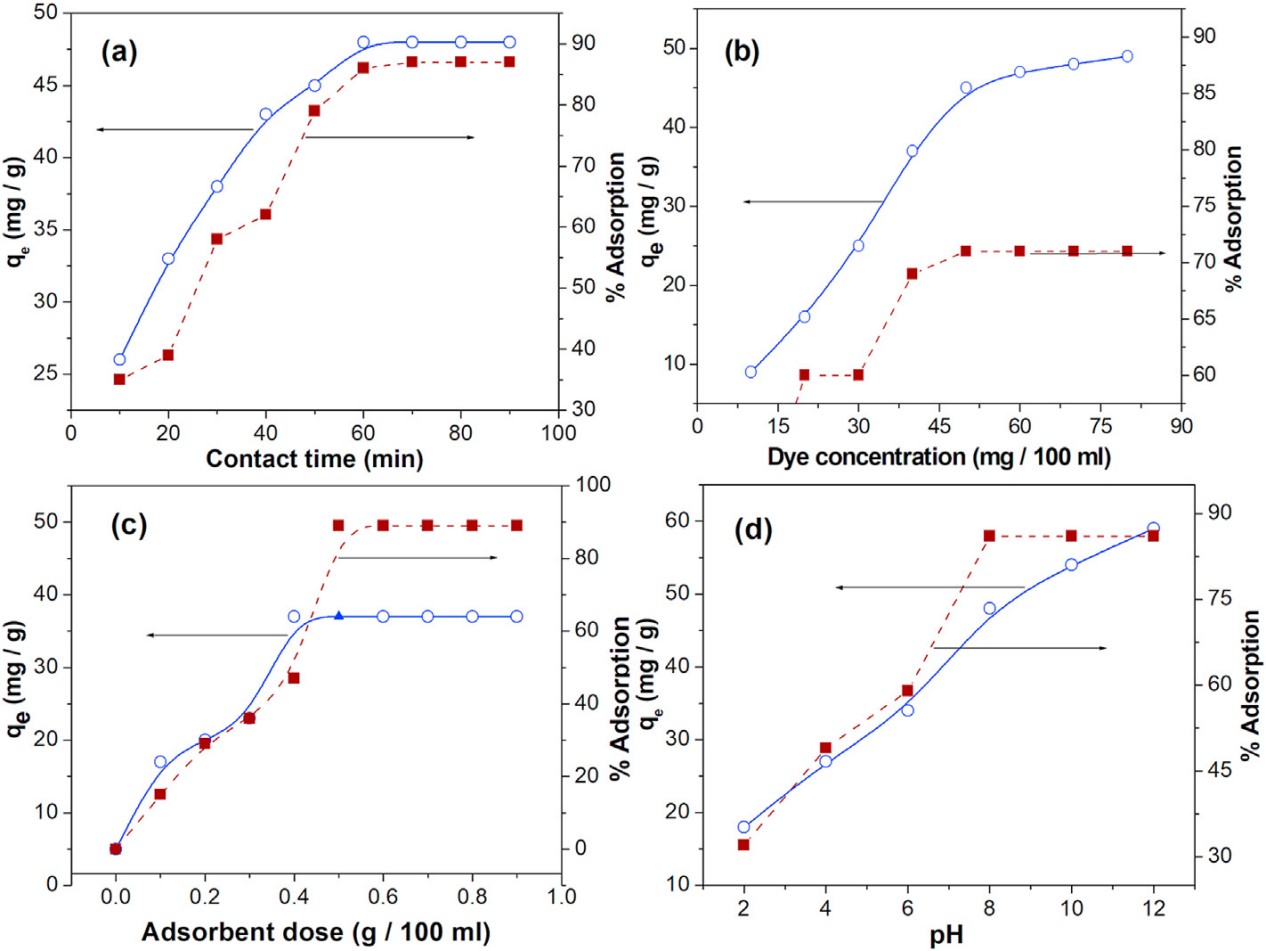
(a) Effect of contact time, (b) Effect of dye concentration, (c) Effect of adsorbent dosage and (d) Effect of pH on RhB dye adsorption.

The impact of contact time at the elimination of RhB dye is depicted in Figure 5a. The dye adsorption of about 85% takes place within of 60 min for RGO–Ni nanocomposite. The equilibrium was attained after 60 min. The rate of adsorption to the maximum extent is due to the vacant pores of adsorbent and gradient solute concentration. The reduced adsorption, in particular towards the end of experiments, suggests the lack of active sites for dye adsorption after completing the equilibrium [30]. Compared to Gelatin/activated carbon composite bead form (dosage 0.15 g/L), zinc oxide loaded activated carbon ZnO-AC (dosage 0.02 g/L) and Ti-doped layered zinc hydroxide (0.3 g/L) which has more contact time of 30 h, 140 min and 240 min, respectively [31, 32, 33]. The present work, RGO–Ni nanocomposite shows higher adsorption capacity and less contact time of 1 h (60 min) due to large surface of graphene.

### 3.2 Effect of dye concentration

The effect of dye concentration on adsorption of RhB onto RGO–Ni nanocomposite was performed in a concentration range of 10–90 mg/100 ml (Figure 5b). Equilibrium adsorption capacity is determined by maintaining an optimum dose of 0.5 g/100 ml for 60 min at a pH of 8. The results indicate that percentage of removal increases with an increase in RhB concentration from 10 mg to 50 mg/100 ml after that adsorption capacity becomes constant [30].

### 3.3 Effect of adsorbent dosage

The effect of adsorbent dose on RhB adsorption is examined by varying the adsorbent dosage from 0.1 – 0.9 g/100 ml. It has been observed from Figure 5c, an increase of the adsorbent dose from 0.1 g/100 ml–0.5 g/100 ml gets a sharp increase in the RhB adsorption. This may be due to the presence of many adsorbent sites which enable greater adsorption and also larger accessibility of surfaces of the adsorbents. Nevertheless, no substantial variations in removal efficiency are noticed beyond 0.5 g/100 ml of adsorbent dose. The reduced adsorption, in the end of experiments, suggests the monolayer formation of RhB and lack of active sites after completing the equilibrium. So, 0.5 g/100 ml is fixed as an optimal dose for RGO–Ni loading where about 90% of RhB adsorption is recorded.

### 3.4 Effect of pH

The pH of the dye solution is one of the important aspects for the adsorption of RhB dye onto RGO–Ni nanocomposite. Figure 5d indicates effect of pH on adsorption to RGO–Ni nanocomposite. The maximum RhB removal is found at the pH 8. The basic dye gives charged ions when basic dye dissolved in water. Hence, in acidic medium, it is observed, in an expanded adsorption of RhB due to increased electrostatic attraction between negatively charged adsorbent and positively charged dye. Beyond pH 8 (alkaline media) adsorbent's positive surface tends to repel cationic adsorbate adsorption. Hence after pH 8.0 adsorption capacity becomes constant [30].

### 3.5 Adsorption isotherm

The adsorption isotherm is an important aspect in the study of adsorptive degradation of dye by the adsorbents. The mechanism and working of adsorbent can be studied by these models. The adsorption isotherms express the quantitative correlation between the dye concentration and the mass of dye adsorbed at a given time, dose, and pH. This experiment enables to calculate greatest adsorption capacity of the adsorbent which depends on mathematical model applied. Langmuir, Freundlich and Temkin models are midst explaining solid-liquid sorption systems. The adsorption capacity and dye concentration in the solution were calculated as per the literature.

**Figure 6:**
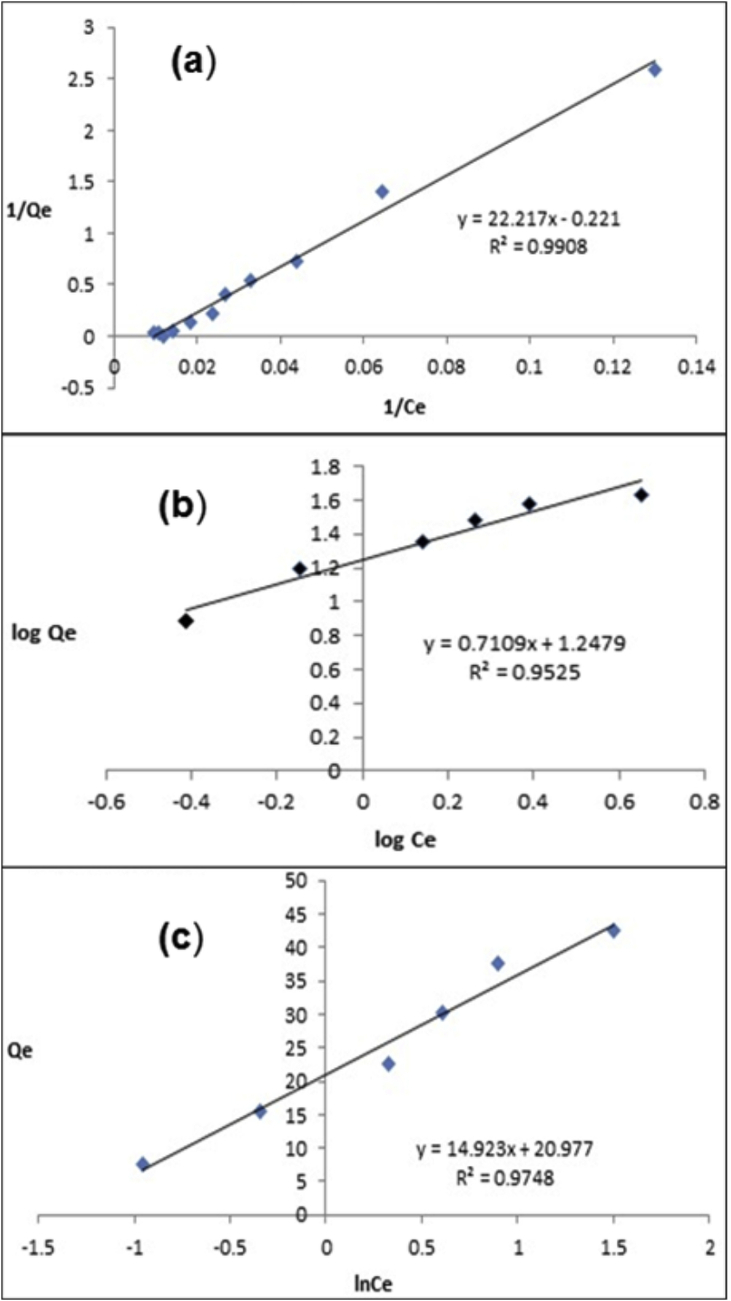
Isotherms (a) Langmuir, (b) Freundlich and (c) Temkin.

Theoretical Langmuir isotherm model is calculated as per literature [34]. Figure 6 shows the Langmuir (Figure 6a), Freundlich (Figure 6b) and Temkin isotherms (Figure 6c). The Langmuir model adopts that the highest adsorption takes in the monolayer of dye primarily on the adsorbent surface and that all the sites have least interaction between adsorbed molecules and the nanomaterial with similar energy. The Freundlich adsorption isotherm is a practical model which can be used in heterogeneous surface system and are calculated as per literature [35]. Temkin isotherm measured as the effects of indirect adsorption heat of all the RhB dye molecules on the RGO–Ni nanocomposite surface will change linearly and results are calculated as per literature [36]. The Langmuir, Freundlich, and Temkin equations were applied to the experimentally determined data (Table 1) and revealed that the Langmuir model fits well (R^2^ = 0.99) than the Freundlich model (R^2^ = 0.95) or the Temkin model (R^2^ = 0.97). On this basis, also one would say that it was chemisorption.

**Table 1:** The adsorption isotherms.

Model	Isothermal Constants			
Langmuir	**q**_**e**_**(mg/g)**	**K**_**L**_**(L/mg)**	**R**_**L**_	**R**^**2**^
	47.62	0.04	0.05–0.35	0.99
Freundlich	**n**	**(mg/g)**	**-**	**R**^**2**^
	2.51	5.69	-	0.95
Tempkin	**(L/g)**	**(mg/L)**	**(J/mg)**	**R**^**2**^
	0.53	9.17	274.66	0.97

### 3.6 Kinetic study of adsorption

**Figure 7:**
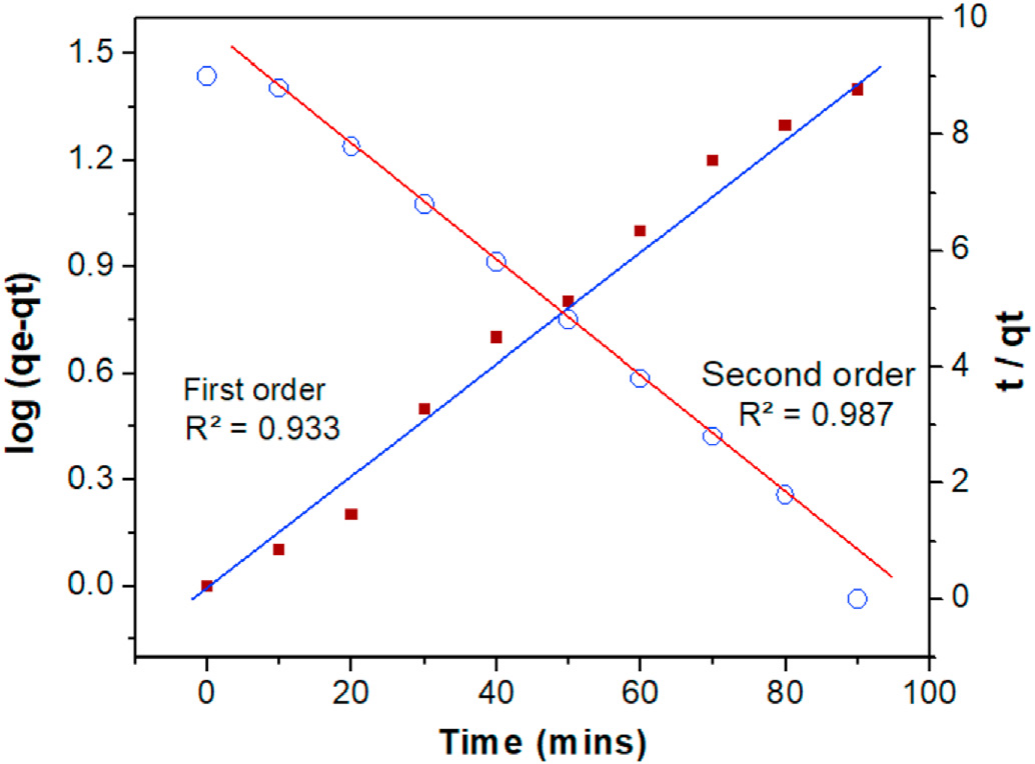
Pseudo 1^st^ and 2^nd^ order plots of adsorption of RhB dye.

The study of kinetics of RhB dye adsorption gives the information about choosing the optimised conditions for the batch process. The kinetic parameters, which are used to study adsorption rate, give insight on about creating and designing the optimised adsorption processes. Different models have been used to assess the adsorption process. Adsorption mechanisms consistent with kinetics-based models have recently been calculated and published in the literature [37, 38]. In this study, pseudo 1^st^ order and Pseudo 2^nd^ order models were used to found the best fitted model for the experimental data obtained (Figure 7). The kinetic study determined that the adsorption of RhB dye follows a pseudo-second order kinetic model. The kinetic constants determined from both the pseudo first order and pseudo second order graphs demonstrate that pseudo second order fits better than pseudo first order (Table 2).

**Table 2:** Kinetics of RhB dye adsorption.

Pseudo 1^st^ order model	**k**_**1**_**(min**^**−1**^**)**	**q**_**e**_**(mg/g)**	**R**^**2**^
	0.0205	65.31	0.933
Pseudo 2^nd^ order model	**k**_**2**_**(g/(mg min**^**−1**^**))**	**q**_**e**_**(mg/g)**	**R**^**2**^
	0.00019	55.56	0.989

### 3.7 Mechanism of adsorption

**Figure 8:**
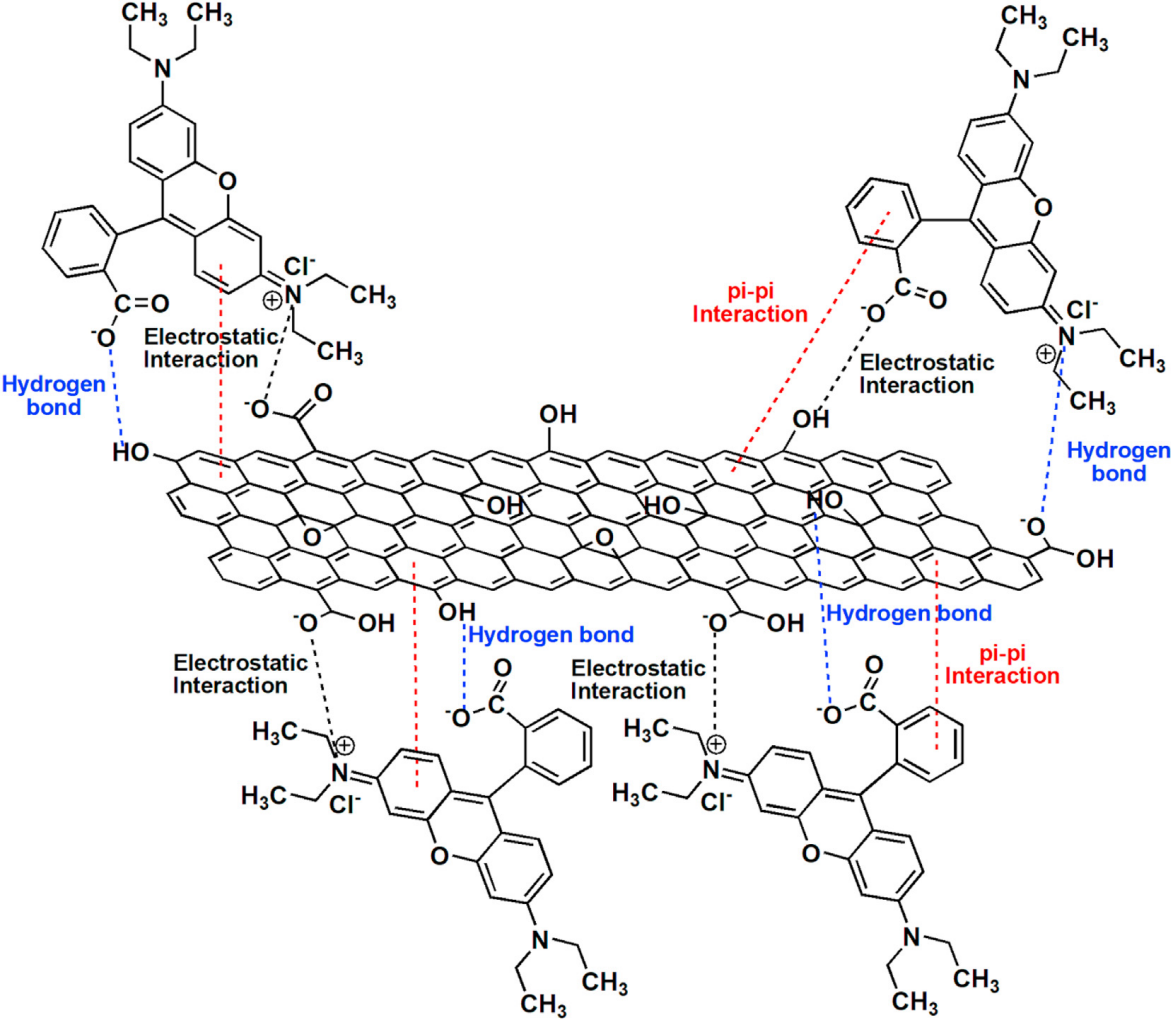
The possible mechanism of interaction between RGO–Ni nanocomposite and Rhodamine blue dye (Blue dashed line indicates Hydrogen bond; Red line indicates π-πinteraction, Black line indicates Electrostatic interaction).

Due to higher specific area of GO it strongly absorbs the dye. In this process it involves three steps, they are (i) the dye molecule moved to the outer surface of adsorbent nanomaterial, (ii) the dye gets into the pores of adsorbent nanomaterial particles and (iii) the adsorption takes place on the surface on the nanomaterial. The electrostatic interactions and hydrogen bonding between the π electrons of GO and the cationic dyes occur during the chemisorption process. RhB is a planar molecule that is readily adsorbed due to the π-π interactions between the dyes' aromatic backbones and the hexagonal skeleton of GO [37, 38], as shown in Figure 8. Thus, it was believed that the Ni-GO composite will play a major role in organic pollutant reduction. As a result, the oxygen functional group in GO enhanced the Ni–GO composite's ability to remove molecular organic contaminants.

### 3.8 Comparison of different adsorbents

A comparison of the maximum adsorption capacity (qm, mg g^−1^) of RhB dye uptake by RGO–Ni nanocomposite to those of other adsorbents reported in the literature is given in Table 3. Many of these polymeric and bioadsorbents have been used to remove RhB dye. The RGO–Ni nanocomposite employed in this study far exceeded a particular adsorption capacity than polymeric, natural and synthetic bio adsorbents.

**Table 3:** Comparison of maximum RhB dye adsorption capacities (qm, mg g^−1^) by different adsorbents reported as per prior literature.

S.No.	Adsorbent	Adsorptive capacity (qm) (mg/g)	Ref.
1	Ligno sulfonate Fe_3_O_4_ Cr(Ⅵ)	22.47	[39]
2	Furfural residue (FR)	37.93	[40]
3	Cal-ZIF-67/AC	46.2	[41]
4	Hyper crosslinked polymeric adsorbent	25–55	[42]
5	Starch grafted p-tert-butyl-calix[n]arene	9.81	[43]
6	Magnetic nanocomposite	29.48	[44]
7	Polyamide branches grafted onto carbon microspheres	19.9	[45]
8	Coconut coir	14.9	[46]
9	Sodium montmorillonite	38.27	[47]
10	RGO-Ni nanocomposite	65.31	Present work

### 3.9 Regeneration and reusability

**Figure 9:**
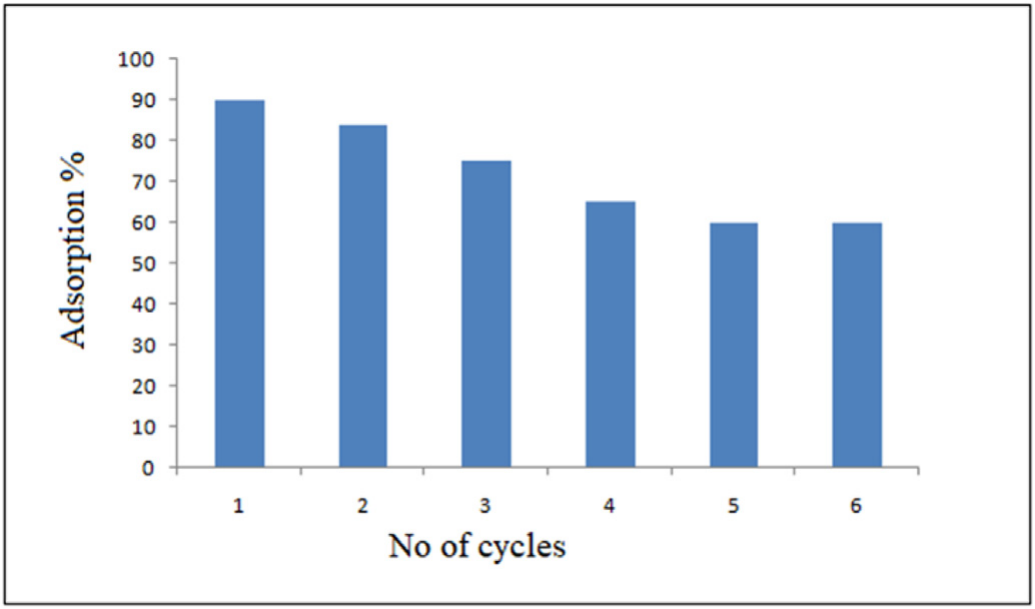
Percentage removal of RhB for regenerated adsorbent at different cycle runs.

We have shown that adsorption is an easy and inexpensive process. Regeneration and reusability are important aspects of the synthesised RGO–Ni nanocomposite, which was put in a beaker and allowed to adsorb in the presence of RhB dye while being vigorously stirred for approximately 60 min. After filtering the adsorbent, it was cleaned with deionized water. The used adsorbent was heated to about 150 °C to activate the adsorbent sites and remove all adsorbed dye. The cycle was repeated 6 times, each with a stirring time of 60 min with RhB dye, and then the absorbance of the filtrate was measured. The results showed to be promising in Figure 9. After the first recycling, the efficiency decreased to 85%, which could be attributed to nanoparticle clustering during sintering. But, for a second round the efficiency decreased as the order was repeated. Therefore, this material can be used for many numbers of times for purification/adsorption.

## 4 Conclusions

In this investigation RGO–Ni nanocomposite suggests favorable adsorption potential of RhB adsorption. The effective parameters optimized for the most sorption are concentration (50 mg/100 ml), sorbent dosage (0.5 g/100 ml) and contact time (60 min). Removal of RhB dye depends on pH and it will be highest at pH 8. Adsorption equilibrium values were fitted nicely inside the Tempkin, Langmuir and Freundlich isotherm models. The rate of sorption determined to obey pseudo-second-order kinetics. The mechanism of the adsorption is defined. The prepared adsorbent is compared with other adsorbents with the same dye and concluded that it is better than other adsorbents reported in literature. Regeneration and reusability study has been done.

## Declarations

### Author Contribution statement

Usha Jinendra: Performed the experiments.

Dinesh Bilehal: Conceived and designed the experiments.

Avvaru Praveen Kumar: Analyzed and interpreted the data; Wrote the paper.

B. M. Nagabhushana: Contributed reagents, materials, analysis tools or data.

### Funding statement

This research did not receive any specific grant from funding agencies in the public, commercial, or not-for-profit sectors.

### Data availability statement

Data included in article/supplementary material/referenced in article.

### Declaration of interests statement

The authors declare no conflict of interest.

### Additional information

No additional information is available for this paper.
